# 4-Bromo-2-((*E*)-{4-[(3,4-dimethyl­isoxazol-5-yl)sulfamo­yl]phen­yl}iminio­meth­yl)phenolate

**DOI:** 10.1107/S160053680800682X

**Published:** 2008-03-14

**Authors:** M. Nawaz Tahir, Zahid H. Chohan, Hazoor A. Shad, Islam Ullah Khan

**Affiliations:** a Department of Physics, University of Sargodha, Sargodha, Pakistan; b Department of Chemistry, Bahauddin Zakariya University, Multan 60800, Pakistan; c Department of Chemistry, Government College University, Lahore, Pakistan

## Abstract

The title compound, C_18_H_16_BrN_3_O_4_S, is a Schiff base ligand of 5-bromo­salicylaldehyde and sulfisoxazole [or *N*-(3,4-dimethyl-5-isoxazol)sulfanilamide]. The present structure is a zwitterion and is a more precise reinterpretation of the structure which was originally reported by Hämäläinen, Lehtinen & Turpeinen [*Arch. Pharm*. (1986), **319**, 415–420]. The two aromatic rings which make π–π inter­actions [centroid–centroid distance 3.7538 (18) Å] through intermolecular interactions. There is also a C—Br⋯π inter­action [3.6333 (15) Å] with the heterocyclic ring. An intra­molecular N—H⋯O hydrogen bond also exists. Dimers are formed due to inter­molecular N—H⋯O hydrogen bonding. Inter­molecular C—H⋯O hydrogen bonding links a methyl C atom and the phenolate O atom. The dimers are linked by C—H⋯N hydrogen bonds, where the C atom is from the Schiff base group and the N atom is of five-membered heterocyclic ring.

## Related literature

For related literature, see: Chohan *et al.* (2008 ▶); Hämäläinen *et al.* (1986 ▶); Shad *et al.* (2008 ▶).
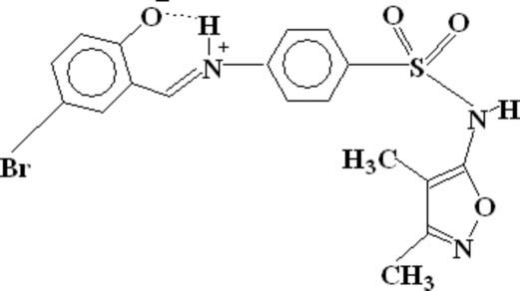


## Experimental

### 

#### Crystal data

C_18_H_16_BrN_3_O_4_S
*M* *_r_* = 450.31
Monoclinic, 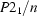

*a* = 15.3846 (10) Å
*b* = 7.2235 (5) Å
*c* = 16.5520 (11) Å
β = 93.201 (4)°
*V* = 1836.6 (2) Å^3^
*Z* = 4
Mo *K*α radiation radiation
μ = 2.38 mm^−1^
*T* = 296 (2) K
0.18 × 0.14 × 0.10 mm

#### Data collection

Bruker Kappa APEXII CCD diffractometer
Absorption correction: multi-scan (*SADABS*; Bruker, 2005 ▶) *T*_min_ = 0.685, *T*_max_ = 0.793
18874 measured reflections
3955 independent reflections
2704 reflections with *I* > 2σ(*I*)
*R*_int_ = 0.058

#### Refinement

*R*[*F* ^2^ > 2σ(*F* ^2^)] = 0.042
*wR*(*F* ^2^) = 0.115
*S* = 1.00
3955 reflections
252 parameters
H atoms treated by a mixture of independent and constrained refinement
Δρ_max_ = 0.46 e Å^−3^
Δρ_min_ = −0.62 e Å^−3^

### 

Data collection: *APEX2* (Bruker, 2007 ▶); cell refinement: *APEX2*; data reduction: *SAINT* (Bruker, 2007 ▶); program(s) used to solve structure: *SHELXS97* (Sheldrick, 2008 ▶); program(s) used to refine structure: *SHELXL97* (Sheldrick, 2008 ▶); molecular graphics: *ORTEP-3 for Windows* (Farrugia, 1997 ▶) and *PLATON* (Spek, 2003 ▶); software used to prepare material for publication: *WinGX* (Farrugia, 1999 ▶) and *PLATON*.

## Supplementary Material

Crystal structure: contains datablocks global, I. DOI: 10.1107/S160053680800682X/bq2069sup1.cif

Structure factors: contains datablocks I. DOI: 10.1107/S160053680800682X/bq2069Isup2.hkl

Additional supplementary materials:  crystallographic information; 3D view; checkCIF report

## Figures and Tables

**Table 1:** Hydrogen-bond geometry (Å, °)

*D*—H⋯*A*	*D*—H	H⋯*A*	*D*⋯*A*	*D*—H⋯*A*
N1—H1⋯O1	0.80 (3)	1.91 (3)	2.577 (4)	141 (3)
N2—H2⋯O1^i^	0.75 (3)	2.09	2.828 (4)	171 (4)
C17—H17*C*⋯O1^i^	0.96	2.58	3.248 (5)	126
C7—H7⋯N3^ii^	0.93	2.53	3.420 (4)	161

Symmetry codes: (i) 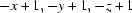; (ii) 
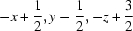
.

**Fig. 1.**
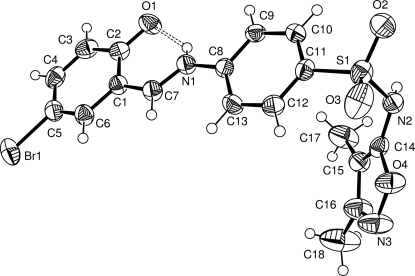
ORTEP-3 for Windows (Farrugia, 1997) drawing of the title compound, C18H16BrN3O4S, with the atom numbering scheme. The thermal ellipsoids are drawn at the 50% probability level. H-atoms are shown by small circles of arbitrary radii. The intramolecular H-bonding is shown by dashed lines.

**Fig. 2.**
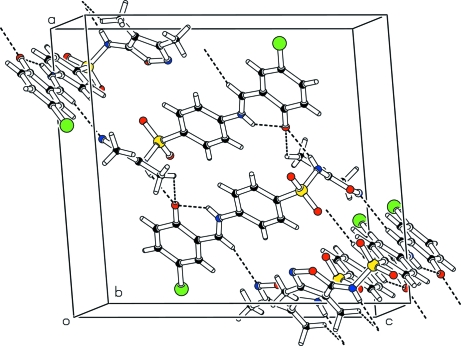
The interaction diagram of (I) (Spek, 2003) showing the intramolecular and intermolecular hydrogen bonding which leads to dimers and a three-dimensional polymeric network.

## supplementary crystallographic information

### Comment

Sulfonamides are a class of compounds, which have been found to possess a wide range of medicinal properties. In continuation of synthesizing Schiff base ligands of substituted halogen salicylaldehyde and various sulfonamides (Chohan *et al.*, 2008, Shad *et al.*, 2008), We report the refinement of (5-Bromosalicy1idene)-*N*-(3,4-dimethyl-5-isoxazolyl)sulfanilamide (Hämäläinen *et al.*, 1986).

During the refinement of 4-Chloro-2-[(*E*)-({4-[(3,4-dimethylisoxazol-5-yl) sulfamoyl]phenyl}iminio)methyl]phenolate (Shad *et al.*, 2008) prepared from 5-chlorosalicylaldehyde and sulfisoxazole: [*N*-(3,4-dimethyl-5-isoxazol) sulfanilamide], it was observed that H-atom of hydroxy group shifts to N-atom of Schiff base moiety. To see the effect of sulfisoxazole on 5-Bromosalicylaldehyde, the title compound (I) has been prepared. The X-ray structure analysis clearly shows that again a zwitterion is formed due to the reaction of 5-Bromosalicylaldehyde and sulfisoxazole. The values of bond lengths and bond angles are similar to the chloro isomer and also similar to the reported by Hämäläinen *et al.*, 1986. There exist π interaction at a distance of 3.6333 (15) Å C5—Br1···CgA^i^ [symmetry code i = *x* - 1/2, -*y* + 1/2, *z* - 1/2], where CgA is the center of gravity of the (A) five-membered heterocyclic ring containing O4. The π - π interaction also exists between the aromatic rings (B) and (C), containing C10 and C3 respectively. The distance between the centroids of neighbouring rings have value of 3.7538 (18) Å, CgB···CgC^ii^ [symmetry code ii = *x*, *y* - 1, *z*], here CgB and CgC represent the center of gravity of ring (B) and ring (C) respectively. The dihedral angles between the rings A/B, A/C, B/C have values of 43.30 (18)°, 44.30 (18)° and 5.56 (15)° respectively. The molecules form dimers through H-bonding between N2—H2···O1^iii^ [symmetry code iii = -*x* + 1, -*y* + 1, -*z* + 1]. These dimers are linked to each other by C7—H7···N3^iv^ [symmetry code iv = -*x* + 1/2, *y* - 1/2, -*z* + 3/2] and form a three-dimensional polymeric network. The intermolecular H-bond [C—H···O] between methyl C-atom and O-atom of aromatic ring with Bromom group. The detail of H-bonding is given in Table 1.

### Experimental

Sulfisoxazole (0.5346 g, 2 mmol) in ethanol (20 ml) was mixed with ethanolic solution (10 ml) of 5-Bromosalicylaldehyde (0.402 g, 2 mmol). After refluxing for 3 h, the solution turned orange red. The solution was cooled to room temperature, filtered and volume reduced to about one-third using rotary evaporator. It was then allowed to stand for 10 days and obtained crystals of title compound. m.p; 481 K.

### Refinement

The coordinates of H-atoms attached to N-atoms were refined. H-atoms were positioned geometrically, with C—H = 0.93 Å for aromatic like, 0.96 Å for methyl and constrained to ride on their parent atoms. The *U*_iso_(H) = *xU*_eq_(C, N), where *x* = 1.5 for methyl H, and *x* = 1.2 for all other H atoms.

### Crystal data

**Table:**

C_18_H_16_BrN_3_O_4_S	*F*_000_ = 912
*M**_r_* = 450.31	*D*_x_ = 1.629 Mg m^−^^3^
Monoclinic, *P*2_1_/*n*	Melting point: 481 K
Hall symbol: -P 2yn	Mo *K*α radiation radiation λ = 0.71073 Å
*a* = 15.3846 (10) Å	Cell parameters from 2704 reflections
*b* = 7.2235 (5) Å	θ = 1.8–27.0º
*c* = 16.5520 (11) Å	µ = 2.38 mm^−^^1^
β = 93.201 (4)º	*T* = 296 (2) K
*V* = 1836.6 (2) Å^3^	Prismatic, red
*Z* = 4	0.18 × 0.14 × 0.10 mm

### Data collection

**Table:**

Bruker KappaAPEXII CCD diffractometer	3955 independent reflections
Radiation source: fine-focus sealed tube	2704 reflections with *I* > 2σ(*I*)
Monochromator: graphite	*R*_int_ = 0.058
Detector resolution: 7.40 pixels mm^-1^	θ_max_ = 27.0º
*T* = 296(2) K	θ_min_ = 1.8º
ω scans	*h* = −19→19
Absorption correction: multi-scan(SADABS; Bruker, 2005)	*k* = −9→9
*T*_min_ = 0.685, *T*_max_ = 0.793	*l* = −21→18
18874 measured reflections	

### Refinement

**Table:**

Refinement on *F*^2^	Secondary atom site location: difference Fourier map
Least-squares matrix: full	Hydrogen site location: inferred from neighbouring sites
*R*[*F*^2^ > 2σ(*F*^2^)] = 0.042	H atoms treated by a mixture of independent and constrained refinement
*wR*(*F*^2^) = 0.115	*w* = 1/[σ^2^(*F*_o_^2^) + (0.00.057*P*)^2^ + 0.7324*P*] where *P* = (*F*_o_^2^ + 2*F*_c_^2^)/3
*S* = 1.01	(Δ/σ)_max_ < 0.001
3955 reflections	Δρ_max_ = 0.46 e Å^−^^3^
252 parameters	Δρ_min_ = −0.62 e Å^−^^3^
Primary atom site location: structure-invariant direct methods	Extinction correction: none

### Special details

**Table:**

Geometry. All e.s.d.'s (except the e.s.d. in the dihedral angle between two l.s. planes) are estimated using the full covariance matrix. The cell e.s.d.'s are taken into account individually in the estimation of e.s.d.'s in distances, angles and torsion angles; correlations between e.s.d.'s in cell parameters are only used when they are defined by crystal symmetry. An approximate (isotropic) treatment of cell e.s.d.'s is used for estimating e.s.d.'s involving l.s. planes.
Refinement. Refinement of *F*^2^ against ALL reflections. The weighted *R*-factor *wR* and goodness of fit *S* are based on *F*^2^, conventional *R*-factors *R* are based on *F*, with *F* set to zero for negative *F*^2^. The threshold expression of *F*^2^ > σ(*F*^2^) is used only for calculating *R*-factors(gt) *etc*. and is not relevant to the choice of reflections for refinement. *R*-factors based on *F*^2^ are statistically about twice as large as those based on *F*, and *R*- factors based on ALL data will be even larger.

### Fractional atomic coordinates and isotropic or equivalent isotropic displacement parameters (Å^2^)

**Table:**

	*x*	*y*	*z*	*U*_iso_*/*U*_eq_	
Br1	0.11987 (2)	−0.44715 (5)	0.41651 (3)	0.05783 (16)	
S1	0.39184 (6)	0.99341 (12)	0.67050 (5)	0.0440 (2)	
O1	0.38204 (15)	0.1394 (3)	0.34949 (14)	0.0491 (6)	
O2	0.4329 (2)	1.1298 (3)	0.62307 (15)	0.0647 (7)	
O3	0.31683 (17)	1.0380 (4)	0.71321 (17)	0.0645 (8)	
O4	0.40342 (15)	0.8167 (3)	0.85228 (14)	0.0514 (6)	
N1	0.32829 (17)	0.3238 (4)	0.47030 (16)	0.0365 (6)	
H1	0.364 (2)	0.300 (5)	0.439 (2)	0.044*	
N2	0.46806 (18)	0.9255 (4)	0.73775 (17)	0.0380 (6)	
H2	0.510 (2)	0.918 (5)	0.717 (2)	0.046*	
N3	0.3987 (2)	0.6529 (5)	0.89824 (19)	0.0669 (10)	
C1	0.26586 (19)	0.0371 (4)	0.42687 (18)	0.0352 (7)	
C2	0.3239 (2)	0.0154 (4)	0.36301 (19)	0.0382 (7)	
C3	0.3146 (2)	−0.1448 (5)	0.3150 (2)	0.0466 (8)	
H3	0.3501	−0.1604	0.2718	0.056*	
C4	0.2547 (2)	−0.2782 (5)	0.33041 (19)	0.0449 (8)	
H4	0.2503	−0.3839	0.2984	0.054*	
C5	0.1997 (2)	−0.2558 (5)	0.3945 (2)	0.0416 (7)	
C6	0.2043 (2)	−0.1014 (5)	0.4411 (2)	0.0407 (8)	
H6	0.1666	−0.0871	0.4826	0.049*	
C7	0.2714 (2)	0.1925 (4)	0.47838 (19)	0.0383 (7)	
H7	0.2330	0.2017	0.5197	0.046*	
C8	0.34005 (19)	0.4812 (4)	0.52001 (18)	0.0329 (7)	
C9	0.39958 (18)	0.6121 (4)	0.49609 (18)	0.0349 (7)	
H9	0.4295	0.5926	0.4495	0.042*	
C10	0.41460 (19)	0.7704 (4)	0.54089 (18)	0.0375 (7)	
H10	0.4549	0.8577	0.5255	0.045*	
C11	0.3682 (2)	0.7976 (4)	0.60991 (18)	0.0361 (7)	
C12	0.3092 (2)	0.6689 (5)	0.63357 (19)	0.0414 (8)	
H12	0.2786	0.6893	0.6797	0.050*	
C13	0.2952 (2)	0.5096 (5)	0.5892 (2)	0.0411 (8)	
H13	0.2558	0.4214	0.6056	0.049*	
C14	0.45153 (18)	0.7779 (4)	0.78862 (17)	0.0346 (7)	
C15	0.4783 (2)	0.6014 (5)	0.7904 (2)	0.0410 (8)	
C16	0.4434 (3)	0.5296 (5)	0.8609 (2)	0.0572 (10)	
C17	0.5315 (3)	0.5017 (5)	0.7328 (3)	0.0692 (13)	
H17A	0.5086	0.3792	0.7242	0.104*	
H17B	0.5906	0.4938	0.7546	0.104*	
H17C	0.5300	0.5672	0.6823	0.104*	
C18	0.4540 (4)	0.3344 (6)	0.8916 (3)	0.0978 (18)	
H18A	0.3996	0.2914	0.9103	0.147*	
H18B	0.4976	0.3314	0.9354	0.147*	
H18C	0.4714	0.2559	0.8486	0.147*	

### Atomic displacement parameters (Å^2^)

**Table:**

	*U*^11^	*U*^22^	*U*^33^	*U*^12^	*U*^13^	*U*^23^
Br1	0.0474 (2)	0.0454 (2)	0.0799 (3)	−0.01217 (17)	−0.00429 (19)	−0.00371 (19)
S1	0.0541 (5)	0.0327 (4)	0.0448 (5)	0.0070 (4)	−0.0016 (4)	−0.0070 (4)
O1	0.0517 (13)	0.0490 (14)	0.0487 (13)	−0.0079 (12)	0.0226 (11)	−0.0059 (11)
O2	0.105 (2)	0.0305 (13)	0.0578 (16)	−0.0076 (14)	−0.0076 (15)	0.0067 (12)
O3	0.0561 (15)	0.0677 (18)	0.0693 (17)	0.0268 (13)	−0.0017 (13)	−0.0284 (14)
O4	0.0569 (14)	0.0549 (15)	0.0449 (13)	0.0158 (12)	0.0248 (12)	−0.0003 (12)
N1	0.0342 (14)	0.0397 (15)	0.0362 (15)	−0.0020 (12)	0.0080 (11)	−0.0048 (12)
N2	0.0384 (14)	0.0388 (15)	0.0372 (15)	−0.0013 (12)	0.0068 (12)	−0.0050 (12)
N3	0.083 (2)	0.062 (2)	0.059 (2)	0.0189 (19)	0.0412 (18)	0.0139 (17)
C1	0.0343 (15)	0.0365 (17)	0.0350 (17)	−0.0005 (13)	0.0044 (13)	−0.0045 (14)
C2	0.0399 (17)	0.0392 (18)	0.0357 (17)	0.0051 (14)	0.0052 (14)	−0.0004 (14)
C3	0.058 (2)	0.046 (2)	0.0358 (18)	0.0073 (17)	0.0095 (16)	−0.0042 (16)
C4	0.056 (2)	0.0372 (18)	0.0403 (19)	0.0041 (16)	−0.0072 (16)	−0.0082 (15)
C5	0.0401 (17)	0.0379 (18)	0.0461 (19)	−0.0026 (14)	−0.0036 (15)	0.0010 (15)
C6	0.0347 (16)	0.0437 (19)	0.0440 (19)	−0.0029 (14)	0.0059 (14)	−0.0044 (15)
C7	0.0359 (16)	0.0411 (18)	0.0384 (17)	−0.0021 (14)	0.0066 (14)	−0.0043 (15)
C8	0.0318 (15)	0.0353 (16)	0.0315 (16)	−0.0006 (12)	0.0007 (12)	−0.0019 (13)
C9	0.0348 (16)	0.0408 (17)	0.0294 (16)	−0.0005 (13)	0.0057 (13)	0.0017 (14)
C10	0.0394 (16)	0.0372 (17)	0.0363 (17)	−0.0053 (14)	0.0058 (14)	0.0014 (14)
C11	0.0398 (16)	0.0340 (17)	0.0338 (17)	0.0032 (13)	−0.0032 (13)	−0.0038 (13)
C12	0.0407 (17)	0.048 (2)	0.0365 (17)	−0.0022 (15)	0.0117 (14)	−0.0057 (15)
C13	0.0414 (17)	0.0431 (18)	0.0399 (18)	−0.0120 (14)	0.0126 (15)	−0.0035 (15)
C14	0.0329 (15)	0.0420 (18)	0.0294 (15)	0.0027 (13)	0.0068 (13)	−0.0095 (14)
C15	0.0448 (17)	0.0390 (18)	0.0405 (18)	−0.0009 (14)	0.0132 (15)	−0.0056 (14)
C16	0.067 (2)	0.049 (2)	0.058 (2)	0.0105 (19)	0.025 (2)	0.0077 (18)
C17	0.096 (3)	0.041 (2)	0.076 (3)	0.005 (2)	0.048 (3)	−0.008 (2)
C18	0.140 (5)	0.066 (3)	0.094 (3)	0.028 (3)	0.065 (3)	0.029 (3)

### Geometric parameters (Å, °)

**Table:**

Br1—C5	1.898 (3)	C6—H6	0.9300
S1—O3	1.424 (3)	C7—H7	0.9300
S1—O2	1.429 (3)	C8—C13	1.385 (4)
S1—N2	1.646 (3)	C8—C9	1.389 (4)
S1—C11	1.760 (3)	C9—C10	1.375 (4)
O1—C2	1.294 (4)	C9—H9	0.9300
O4—C14	1.350 (3)	C10—C11	1.395 (4)
O4—N3	1.411 (4)	C10—H10	0.9300
N1—C7	1.302 (4)	C11—C12	1.371 (4)
N1—C8	1.409 (4)	C12—C13	1.376 (5)
N1—H1	0.80 (3)	C12—H12	0.9300
N2—C14	1.391 (4)	C13—H13	0.9300
N2—H2	0.75 (4)	C14—C15	1.339 (4)
N3—C16	1.302 (5)	C15—C16	1.410 (5)
C1—C6	1.406 (4)	C15—C17	1.478 (5)
C1—C7	1.409 (4)	C16—C18	1.504 (6)
C1—C2	1.429 (4)	C17—H17A	0.9600
C2—C3	1.406 (5)	C17—H17B	0.9600
C3—C4	1.368 (5)	C17—H17C	0.9600
C3—H3	0.9300	C18—H18A	0.9600
C4—C5	1.402 (5)	C18—H18B	0.9600
C4—H4	0.9300	C18—H18C	0.9600
C5—C6	1.355 (5)		
			
O3—S1—O2	120.84 (18)	C9—C8—N1	116.6 (3)
O3—S1—N2	107.36 (15)	C10—C9—C8	120.4 (3)
O2—S1—N2	104.87 (16)	C10—C9—H9	119.8
O3—S1—C11	108.49 (16)	C8—C9—H9	119.8
O2—S1—C11	108.95 (16)	C9—C10—C11	118.7 (3)
N2—S1—C11	105.22 (14)	C9—C10—H10	120.7
C14—O4—N3	107.1 (2)	C11—C10—H10	120.7
C7—N1—C8	126.3 (3)	C12—C11—C10	121.1 (3)
C7—N1—H1	114 (3)	C12—C11—S1	120.1 (2)
C8—N1—H1	119 (3)	C10—C11—S1	118.7 (2)
C14—N2—S1	119.4 (2)	C11—C12—C13	120.0 (3)
C14—N2—H2	114 (3)	C11—C12—H12	120.0
S1—N2—H2	108 (3)	C13—C12—H12	120.0
C16—N3—O4	105.9 (3)	C12—C13—C8	119.7 (3)
C6—C1—C7	119.0 (3)	C12—C13—H13	120.2
C6—C1—C2	119.9 (3)	C8—C13—H13	120.2
C7—C1—C2	121.0 (3)	C15—C14—O4	111.2 (3)
O1—C2—C3	121.4 (3)	C15—C14—N2	132.6 (3)
O1—C2—C1	121.2 (3)	O4—C14—N2	116.1 (3)
C3—C2—C1	117.4 (3)	C14—C15—C16	103.8 (3)
C4—C3—C2	121.5 (3)	C14—C15—C17	129.1 (3)
C4—C3—H3	119.3	C16—C15—C17	127.2 (3)
C2—C3—H3	119.3	N3—C16—C15	112.1 (3)
C3—C4—C5	120.1 (3)	N3—C16—C18	122.1 (3)
C3—C4—H4	120.0	C15—C16—C18	125.8 (3)
C5—C4—H4	120.0	C15—C17—H17A	109.5
C6—C5—C4	120.7 (3)	C15—C17—H17B	109.5
C6—C5—Br1	120.2 (3)	H17A—C17—H17B	109.5
C4—C5—Br1	119.0 (3)	C15—C17—H17C	109.5
C5—C6—C1	120.3 (3)	H17A—C17—H17C	109.5
C5—C6—H6	119.8	H17B—C17—H17C	109.5
C1—C6—H6	119.8	C16—C18—H18A	109.5
N1—C7—C1	122.4 (3)	C16—C18—H18B	109.5
N1—C7—H7	118.8	H18A—C18—H18B	109.5
C1—C7—H7	118.8	C16—C18—H18C	109.5
C13—C8—C9	120.1 (3)	H18A—C18—H18C	109.5
C13—C8—N1	123.3 (3)	H18B—C18—H18C	109.5
			
O3—S1—N2—C14	56.9 (3)	C9—C10—C11—S1	176.4 (2)
O2—S1—N2—C14	−173.4 (2)	O3—S1—C11—C12	−29.4 (3)
C11—S1—N2—C14	−58.5 (3)	O2—S1—C11—C12	−162.7 (3)
C14—O4—N3—C16	0.5 (4)	N2—S1—C11—C12	85.3 (3)
C6—C1—C2—O1	−178.9 (3)	O3—S1—C11—C10	154.8 (3)
C7—C1—C2—O1	−1.4 (5)	O2—S1—C11—C10	21.5 (3)
C6—C1—C2—C3	1.8 (5)	N2—S1—C11—C10	−90.5 (3)
C7—C1—C2—C3	179.3 (3)	C10—C11—C12—C13	0.2 (5)
O1—C2—C3—C4	178.3 (3)	S1—C11—C12—C13	−175.5 (3)
C1—C2—C3—C4	−2.3 (5)	C11—C12—C13—C8	−0.9 (5)
C2—C3—C4—C5	1.0 (5)	C9—C8—C13—C12	0.7 (5)
C3—C4—C5—C6	0.9 (5)	N1—C8—C13—C12	−178.7 (3)
C3—C4—C5—Br1	−177.9 (3)	N3—O4—C14—C15	−0.2 (4)
C4—C5—C6—C1	−1.4 (5)	N3—O4—C14—N2	−176.6 (3)
Br1—C5—C6—C1	177.4 (2)	S1—N2—C14—C15	105.7 (4)
C7—C1—C6—C5	−177.5 (3)	S1—N2—C14—O4	−78.9 (3)
C2—C1—C6—C5	0.0 (5)	O4—C14—C15—C16	−0.1 (4)
C8—N1—C7—C1	−178.3 (3)	N2—C14—C15—C16	175.5 (4)
C6—C1—C7—N1	178.1 (3)	O4—C14—C15—C17	179.7 (4)
C2—C1—C7—N1	0.6 (5)	N2—C14—C15—C17	−4.7 (7)
C7—N1—C8—C13	4.9 (5)	O4—N3—C16—C15	−0.6 (5)
C7—N1—C8—C9	−174.5 (3)	O4—N3—C16—C18	−180.0 (4)
C13—C8—C9—C10	0.1 (5)	C14—C15—C16—N3	0.5 (5)
N1—C8—C9—C10	179.6 (3)	C17—C15—C16—N3	−179.4 (4)
C8—C9—C10—C11	−0.8 (5)	C14—C15—C16—C18	179.8 (5)
C9—C10—C11—C12	0.6 (5)	C17—C15—C16—C18	0.0 (7)

### Hydrogen-bond geometry (Å, °)

**Table:**

*D*—H···*A*	*D*—H	H···*A*	*D*···*A*	*D*—H···*A*
N1—H1···O1	0.80 (3)	1.91 (3)	2.577 (4)	141 (3)
N2—H2···O1^i^	0.75 (3)	2.09	2.828 (4)	171 (4)
C17—H17C···O1^i^	0.96	2.58	3.248 (5)	126
C7—H7···N3^ii^	0.93	2.53	3.420 (4)	161

Symmetry codes:  (i) −*x*+1, −*y*+1, −*z*+1; (ii) −*x*+1/2, *y*−1/2, −*z*+3/2.
